# Ru and W isotope systematics in ocean island basalts reveals core leakage

**DOI:** 10.1038/s41586-025-09003-0

**Published:** 2025-05-21

**Authors:** Nils Messling, Matthias Willbold, Leander Kallas, Tim Elliott, J. Godfrey Fitton, Thomas Müller, Dennis Geist

**Affiliations:** 1 Department for Geochemistry and Isotope Geology, Georg-August-Universität Göttingen, Göttingen, Germany; 2 Bristol Isotope Group, School of Earth Sciences, Wills Memorial Building, Queen’s Road, University of Bristol, Bristol, UK; 3 School of GeoSciences, University of Edinburgh, Grant Institute, James Hutton Road, Edinburgh, UK; 4 Geoscience Center, Georg-August-Universität Göttingen, Göttingen, Germany; 5 Geology Department, Colgate University, Hamilton, NY USA

**Keywords:** Geochemistry, Geochemistry

## Abstract

The isotopic composition of lavas associated with mantle plumes has previously been interpreted in the light of core–mantle interaction, suggesting that mantle plumes may transport core material to Earth’s surface^1–5^. However, a definitive fingerprint of Earth’s core in the mantle remains unconfirmed. Precious metals, such as ruthenium (Ru), are highly concentrated in the metallic core but extremely depleted in the silicate mantle. Recently discovered mass-independent Ru isotope variations (*ε*^100^Ru) in ancient rocks show that the Ru isotope composition of accreted material changed during later stages of Earth’s growth^6^, indicating that the core and mantle must have different Ru isotope compositions. This illustrates the potential of Ru isotopes as a new tracer for core–mantle interaction. Here we report Ru isotope anomalies for ocean island basalts. Basalts from Hawaii have higher *ε*^100^Ru than the ambient mantle. Combined with unradiogenic tungsten (W) isotope ratios, this is diagnostic of a core contribution to their mantle sources. The combined Ru and W isotope systematics of Hawaiian basalts are best explained by simple core entrainment but addition of core-derived oxide minerals at the core–mantle boundary is a possibility.

## Abstract (web summary)

Ru isotopes are proposed as tracers for core–mantle interaction on Earth, and anomalies for ocean island basalts from Hawaii are reported that have higher *ε*^100^Ru than the ambient mantle.

## Main

Ocean island basalts (OIB) form by decompression melting of rising, hot mantle plumes. Some deep-rooted mantle plumes possibly originate from the core–mantle boundary (CMB) and are often marked by the striking occurrence of negative *μ*^182^W (parts-per-million (ppm) deviation of ^182^W/^184^W from terrestrial standards)^1,2,5^. Variations in *μ*^182^W could only have been established through the decay of the extinct radionuclide ^182^Hf (*t*_1/2_ = 8.9 million years^7^) following chemical fractionation of Hf and W within the first 60 million years (Myr) of Solar System history (that is, more than 4.5 billion years ago). Some OIB with negative *μ*^182^W have high ^3^He/^4^He ratios^8^, characteristics that could be explained by incorporating material from an isolated, undegassed reservoir formed during the lifetime of ^182^Hf (ref. ^5^). The core formed with low Hf/W ratios, thus retaining a low *μ*^182^W value throughout Earth’s history^1,2^. Together with the high solubility of noble gases in the core during its formation, this would support a core origin of low *μ*^182^W and high ^3^He/^4^He in OIB^2,3,9,10^. Yet, alternative models have been proposed that conform with the chemical and isotopic variations observed in OIB, including the incorporation of material from stranded, late-accreted meteorites in the lower mantle^8,11^ or from different, early-formed silicate reservoirs^5,12,13^.

Highly siderophile element (HSE) concentrations in the mantle are most susceptible to core–mantle interaction because the contrast in HSE concentrations between the core and mantle is several orders of magnitude. A collateral effect of core entrainment at the CMB would be the strong enrichment of HSE and the potential influence on HSE isotope systematics on the lower mantle (for example, ^186^Os/^188^Os and ^187^Os/^188^Os). However, no such effects on HSE systematics can be clearly identified in OIB associated with negative *μ*^182^W (refs. ^14,15^), To reconcile these discrepancies, recent revisions on the core–mantle interaction hypothesis invoke models that could decouple W and He from HSE systematics. The latter require element diffusion across the CMB^10,16,17^, isotopic equilibration between the outer core and the lower mantle^1,18^ or the exsolution of minerals from the outer core^2,3^.

Nucleosynthetic isotope variations of the HSE ruthenium provide a more direct and thus much more powerful tool to investigate the nature of core–mantle interaction. Ruthenium was almost entirely removed from the mantle into the core during Earth’s main accretion^19^. Its budget in the mantle was later replenished through the addition of chondritic material during a late accretionary phase after core formation had ceased^6,20^. Differences in the Ru isotopic composition of modern and ancient mantle-derived rocks suggest that the late-accreted material was compositionally distinct from Earth’s main building blocks^6^. This isotopic disparity is based on the variable contribution of Ru nuclides produced by slow neutron capture (s-process) in different meteorite groups. As the late accretion of meteoritic material does not affect the core, the core should share the s-process-enriched nature of Earth’s earlier, main building blocks. As such we expect mantle sources that record core–mantle interaction to be enriched in s-process Ru nuclides.

Here we determined the Ru isotopic composition of a set of oceanic basalts and picrites from Hawaii and continental picrites from Baffin Island, which have been characterized for their W and He isotope composition in previous studies^1,5,8,21,22^. In addition, we provide new W and Ru isotope data from basalts from Kauai and Kama’ehuakanaloa (Loihi) and basalts associated with the Galápagos and La Réunion hotspots. To better constrain the composition of the upper mantle we included a set of Phanerozoic peridotites (Eifel) and continental picrites (Rhenish massif). We provide additional data for an Eoarchean dunite from Isua (Greenland) and ores from the Bushveld Complex, that have been previously constrained for their Ru isotopic compositions^6,13^, to independently verify our analytical setup. We mainly focus on the ^100^Ru/^101^Ru and ^102^Ru/^101^Ru ratios to constrain Ru s-process variations. These ratios have the largest isotope variability and the highest measurement precision and are therefore the most diagnostic tool for detecting a core contribution in OIB. The variations are reported as *ε*^100^Ru and *ε*^102^Ru, whereby *ε* denotes the 0.01% deviation from a laboratory standard.

## Ru isotopic compositions of OIB

**Fig. 1:**
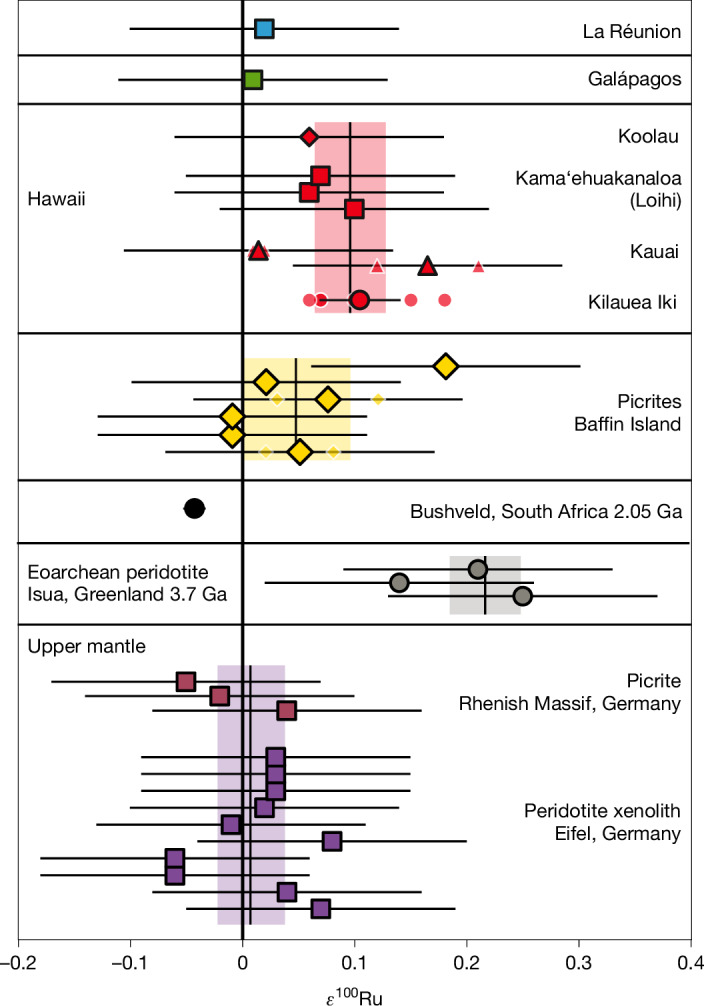
*ε*^100^Ru values of OIBs, picrites and peridotites from the modern and Archean upper mantle. The uncertainties of individual measurements (*n* < 3) were estimated by the 2*σ* of repeated analysis of Bushveld pyroxenite OREAS 684 (*ε*^100^Ru = −0.04 ± 0.13, 2*σ* *n* = 72,  Methods). The error bars represent the external reproducibility 2*σ*. For samples analysed repeatedly (*n* ≥ 4) error bars show the 95% confidence intervals (CI) of replicate analyses. Small symbols with white outlines represent individual measurements for samples that have been analysed repeatedly. *ε*^100^Ru of the modern upper mantle is indicated by the light purple area and defined by the 95% CI of combined data for modern, non-plume-related picrites and peridotites. The light grey area indicates the composition of the Eoarchean mantle^6^. Light yellow and red areas represent the 95% CI of combined Baffin Island and Hawaii data, respectively. Ga, billion years ago. Source data

**Extended Data Fig. 1:**
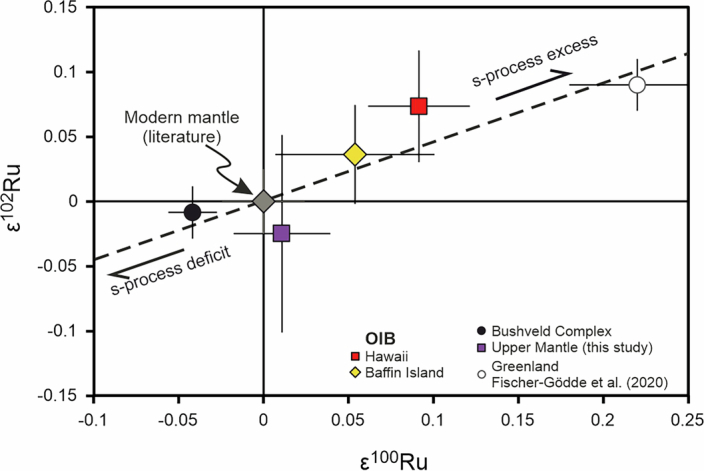
Isotope plot for ε^100^Ru and ε^102^Ru illustrating variations in s-process Ru. The solid line indicates the expected correlation between ε^100^Ru and ε^102^Ru following variable depletions or excesses in s-process Ru. The slope is based on data for pre-solar SiC grains^68^. Literature data for southwest Greenland and the modern mantle are added for comparison^6^. Upper mantle value from this study represents combined data from Eifel peridotites and Rhenish Massif. Due to the large uncertainty of individual measurements, only average data for sample locations with multiple measurements is provided. Error bars are defined by the 95% CI of each group. Source data

The Ru isotope composition of samples and reference materials are provided in Fig. 1, Extended Data Table 1 and Supplementary Table 1, together with *μ*^182^W data. Eifel peridotites and Rhenish picrites measured in this study have averaged *ε*^100^Ru values of 0.02 ± 0.03 and −0.01 ± 0.13 as well as *ε*^102^Ru values of −0.04 ± 0.06 and 0.04 ± 0.18, respectively (details on the estimation of uncertainties are provided in the caption of Fig. 1). Their isotopic compositions are in excellent agreement with values previously published for the modern mantle (*ε*^100^Ru = 0.00 ± 0.02 and *ε*^102^Ru = 0.00 ± 0.02, ref. ^6^; *ε*^100^Ru = 0.01 ± 0.07 and *ε*^102^Ru = 0.01 ± 0.42, ref. ^23^). On the other hand, the averaged composition of Hawaiian picrites and basalts shows a resolvable Ru isotope anomaly with *ε*^100^Ru of 0.09 ± 0.03. Although *ε*^102^Ru values of 0.07 ± 0.04 are not fully resolved from the upper mantle values, combined ^100^Ru and ^102^Ru systematics are in excellent agreement with those expected for nucleosynthetic isotope anomalies, indicating that the Hawaiian plume source is enriched in s-process Ru (Extended Data Fig. 1). Furthermore, two samples from the Kilauea Iki lava lake (*ε*^100^Ru = 0.11 ± 0.04) and the Napali Member on Kauai (*ε*^100^Ru = 0.17 ± 0.13) show excesses in s-process Ru for individual samples. The Hawaiian mafic rocks therefore provide evidence for the presence of s-process-enriched components in the modern mantle. The averaged Ru isotopic composition for high ^3^He/^4^He picrites from Baffin Island shows no resolvable deviations from the upper mantle, with an *ε*^100^Ru value of 0.05 ± 0.05 and *ε*^102^Ru of 0.04 ± 0.04. One individual sample has an elevated *ε*^100^Ru value of 0.18 ± 0.13, indicating a minor excess in s-process Ru within the Baffin Island mantle source. The Ru isotopic compositions of two more samples, associated with the La Réunion and Galápagos Plumes, are not resolvable from the modern upper mantle (*ε*^100^Ru = 0.02 ± 0.13 and 0.01 ± 0.13, respectively). Ru isotope data for an Eoarchean dunite from Isua show a *ε*^100^Ru = 0.20 ± 0.13 (*n* = 3), in excellent agreement with data previously reported for the same locality (*ε*^100^Ru = 0.22 ± 0.04, *n* = 14)^6,13^.

## The Ru isotopic composition of the core

For reasons outlined earlier, a contribution of Ru from the core is an attractive means of generating the *ε*^100^Ru anomalies reported in our OIB. To investigate the viability of this process in more detail it is first important to constrain the *ε*^100^Ru of the core. The Ru isotopic composition of Earth’s mantle very dominantly reflects the late-accreted material, added after core formation had ceased^6,20,24^. By contrast, the composition of bulk Earth, and thus its core, integrates Ru added throughout its history of accretion. As such, mantle and core will have different Ru isotopic compositions, if the material added through late accretion differs from that, added during main accretion.

**Fig. 2:**
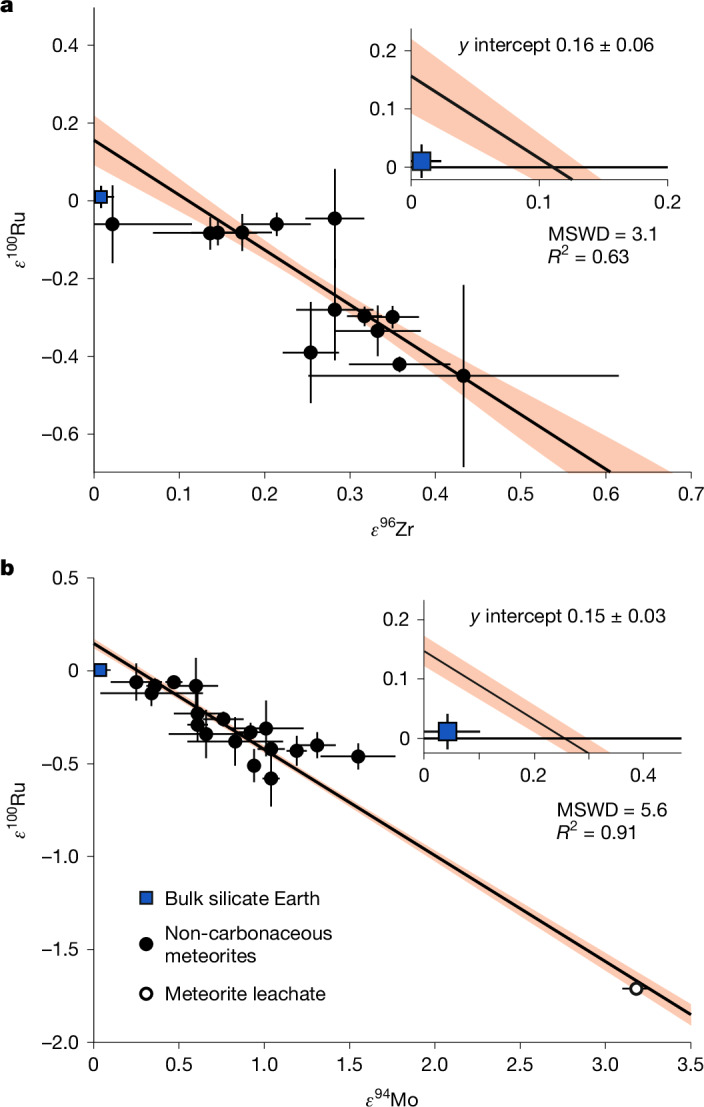
Mo–Ru and Zr–Ru s-process variability in non-carbonaceous meteorites and the bulk silicate Earth. **a**, *ε*^96^Zr and *ε*^100^Ru data originally reported in ref. ^26^. **b**, *ε*^94^Mo and *ε*^100^Ru data originally reported in ref. ^43^. The linear regressions, error envelopes and mean square weighted deviations (MSWD) were calculated using the York method in IsoplotR^44^ based on the meteorite data. The error bars show the 2*σ* measurement uncertainty for *ε*^94^Mo and 95% CI for *ε*^96^Zr and *ε*^100^Ru. The *ε*^100^Ru value and error for the bulk silicate Earth are based on the Eifel peridotite and Hessian picrite data from this study. The inset plots show that bulk silicate Earth does not plot on the non-carbonaceous chondrite correlation defined by either isotope pair. Source data

An estimate of the Earth’s bulk *ε*^100^Ru (and hence that of the core) can be obtained from meteoritic arrays of isotope ratios of other elements influenced by the same nucleosynthetic processes. Similar to Ru, isotopes of Zr and Mo show s-process variability and non-carbonaceous chondrites show clear correlations between the s-process influenced isotope ratios of *ε*^96^Zr, *ε*^94^Mo and *ε*^100^Ru (Fig. 2)^25–27^. As a non-carbonaceous body, bulk Earth is expected to lie on these arrays^25–27^. The Mo and Zr isotope ratios of the silicate Earth sample different phases of accretion compared to Ru. The mantle composition of the moderately siderophile Mo represents the last 10–15% of main accretion, whereas the mantle composition of the lithophile element Zr averages the entire inventory of accretion^24^. Thus the *ε*^100^Ru predicted by the non-carbonaceous meteorite correlation at bulk Earth *ε*^96^Zr (*ε*^96^Zr ≈ 0) should represent the value for the Earth’s core (Fig. 2a). Yet, Earth’s modern silicate mantle plots below this correlation (inset in Fig. 2a), implying that material added during the late accretion lowered its *ε*^100^Ru. Similarly, the *ε*^100^Ru–*ε*^94^Mo composition of Earth’s mantle plots below the correlation defined by non-carbonaceous meteorites (Fig. 2b). Combined, both isotope systems show the predicted bulk Earth, and therefore the core, should have an excess in s-process Ru (*ε*^100^Ru ≈ 0.15, Fig. 2) relative to the modern mantle. This makes the core a suitable mixing component to account for the elevated *ε*^100^Ru of Hawaiian OIB.

## The nature of core–mantle interaction

**Fig. 3:**
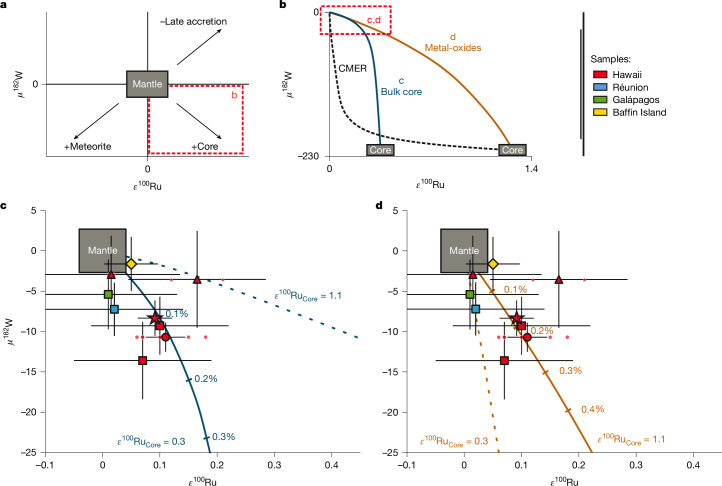
Core–mantle interaction models based on *ε*^100^Ru and *μ*^182^W data of OIB, illustrating the addition of core-related components to the mantle. **a**, Schematic of the effects of core addition, meteorite addition or subtraction of late-accreted material^6^ on the composition of the mantle. **b**, Overview of different core–mantle interaction models. The black dashed line indicates a mixing trend between a CMER and the ambient mantle. Alternative scenarios for the addition of ‘bulk core’ and ‘metal oxides’ are shown in detail in **c** and **d**, respectively. **c**, Binary mixing between bulk core and the mantle. The *ε*^100^Ru composition of the core was chosen to fit Hawaiian OIB data. **d**, Binary mixing between metal oxide-rich outer core layer and the mantle. The composition of the core was calculated by subtracting a late accretion component from the mantle and assuming that the core has a higher *ε*^100^Ru than the PLAM (main text). Less than 0.3% of this oxide-rich core component is required to explain W and Ru isotope variability in OIB. Note that if the *ε*^100^Ru of the core is 0.3, admixing of an oxide-rich core component cannot reproduce the measured data (dashed line) The *μ*^182^W value for Baffin Island is based on the average of data provided in refs. ^17,45^. Error bars show the external reproducibility as 2*σ* for *μ*^182^W. Error bars for *ε*^100^Ru are defined in Fig. 1. The composition of the mantle is defined by the 95% CI of measured reference materials. The star symbol represents the average Hawaiian OIB. For more detailed information on other sample symbols, see Fig. 1. Source data

We evaluate a variety of core–mantle interaction models to account for the combined *ε*^100^Ru–*μ*^182^W OIB data (Fig. 3). In the most straightforward model, bulk core is added to a lower mantle source. Concentrations of Ru and W, as well as the *μ*^182^W of the core are well-constrained^28,29^. We can independently estimate the *ε*^100^Ru composition of the core by fitting a core–mantle mixing curve through the OIB data (Fig. 3b,c). The addition of a bulk core component with *ε*^100^Ru = 0.25–0.35 best describes the W and Ru isotope variability in the OIB data. Despite the simplified nature of this model, the *ε*^100^Ru of the core predicted by the OIB data only slightly exceeds the value derived from the *ε*^100^Ru–ε^96^Zr correlation described in the previous section (*ε*^100^Ru = 0.16 ± 0.06, Fig. 2a). To reconcile the global W isotope variability for OIB (*μ*^182^W = 0 to −20), less than 0.25% of a bulk core component is required. This would result in OIB sources with the lowest *μ*^182^W having a 2.5-fold higher HSE concentrations than those with *μ*^182^W = 0 (Extended Data Table 2). At face value, this may be incompatible with the absence of increased HSE concentrations in OIB with negative *ε*^182^W (ref. ^1^). However, modelling the behaviour of HSE in magmatic systems is very complex. Minor sulfide and alloy phases have a lot of control over partitioning but are poorly constrained and potentially variable between different settings^30–32^. As such, their magmatic behaviour may obscure the effects of core–mantle interaction on HSE concentrations of the mantle source. We favour this simple core–mantle mixing model, but recognizing the potential problem with HSE abundances and also explore an alternative.

To reduce the impact on the HSE concentrations of a plume mantle source, core–mantle interaction must then entail a process that lowers the HSE abundances in the component incorporated into the mantle. A viable model invokes the formation of an oxygen-rich outer core domain and subsequent crystallization of metal-rich oxides through secular core cooling^2,33^. Experimental data indicate that W is enriched in FeO-rich regions of quenched metal alloys whereas HSE are generally depleted^2^. Data for element partitioning between oxygen-rich and oxygen-poor metallic liquids confirm that W prefers oxygen-rich metal alloys whereas the HSE show oxygen-avoiding behaviour^34^. We use this partitioning data to approximate the composition of a metal oxide layer on the outer core to have Ru/W = 1.2.

By reducing the Ru/W of the core component, we consequently need to increase its *ε*^100^Ru to generate a core–mantle mixing array that passes through our data (Fig. 3b,d). In this context, we note that the existence of terrestrial building blocks with *ε*^100^Ru > 0 is required by the occurrence of positive *ε*^100^Ru values in Eoarchean rocks from Greenland (*ε*^100^Ru = 0.22 ± 0.04)^6^. The elevated *ε*^100^Ru in these rocks is thought to represent a mantle domain that has not fully incorporated late-accreted material. By subtraction of a chondritic late veneer from the mantle source of these Greenland samples, Fischer-Gödde et al.^6^ calculated the *ε*^100^Ru of a prelate accretion mantle (PLAM). This estimate strongly depends on the composition of the late veneer, resulting in a large range of PLAM compositions with elevated *ε*^100^Ru = 0.31–3.55 (ref. ^6^). If we assume that the s-process excess of accreting material was constant throughout main accretion, the Ru isotopic composition of the PLAM approximates that of the core. From this perspective, we explore metal oxide-mantle mixing calculations using *ε*^100^Ru = 1.1 for the core as an illustrative and sufficiently high, intermediate value within the range of estimates for PLAM^6^.

As shown in Fig. 3d, the addition of 0.3% of such an oxide-rich outer core layer to the mantle can reproduce the combined Ru and W isotope systematics in OIB. In contrast to the simple core–mantle mixing model above, this process would increase the HSE concentration of the OIB mantle source by only 3–40%. (Extended Data Table 2), which would be difficult to detect in the composition of its erupted melts. We note, however, that a core *ε*^100^Ru = 1.1 is much higher than predicted by the extrapolations of the meteoritic *ε*^100^Ru–*ε*^94^Mo and *ε*^100^Ru–*ε*^96^Zr arrays (Fig. 2), which we argue is probably a stronger constraint than the absence of clear HSE concentration anomalies in the OIB samples with elevated *ε*^100^Ru. Similarly, a core–mantle equilibrated reservoir (CMER^1^), previously proposed to reconcile the lack of HSE enrichment in OIB with a putative core contribution, cannot reconcile the presence of elevated *ε*^100^Ru in Hawaiian OIB. For instance, a CMER composition has been modelled using metal-silicate partition coefficients of HSE and W extrapolated to CMB conditions (Extended Data Table 2)^35,36^. However, incorporation of a CMER (Ru/W < 0.03) will lead to imperceptible changes in the Ru composition in the mantle source regardless of the *ε*^100^Ru value chosen for the core (Fig. 3b, dashed line).

Alternative models not invoking interaction between Earth’s core and mantle have also been suggested to explain observed negative *μ*^182^W. Negative *μ*^182^W may characterize an early enriched silicate reservoir, evolving with low Hf/W (ref. ^37^). In a Hadean, enriched mantle reservoir, for example, *μ*^182^W would be expected to correlate with *μ*^142^Nd because fractionation of Hf/W and Sm/Nd are tightly linked during silicate differentiation^28^, but negative *μ*^142^Nd have not been observed for any plume-related rocks so far^38–40^. Potentially, *μ*^142^Nd and *μ*^182^W systematics may be decoupled during the formation and differentiation of Hadean proto-crust^13^. However, the influence of Hadean recycled crust on the *ε*^100^Ru signature would be negligible because Ru is strongly depleted in the crust compared to the mantle (Archean upper continental crust 0.51 ng g^−1^, ref. ^41^; mantle 7.4 ng g^−1^ Ru, ref. ^42^). On the other hand, negative *μ*^182^W in OIB have also been interpreted to reflect remnants of late, impactor core material preserved in the mantle^8,11^. The incorporation of core material derived from an s-process-enriched late impactor, stranded in the mantle, could hypothetically explain the coupled isotope systematics of Hawaiian OIBs. Yet, the last impactors incorporated into Earth’s mantle as part of the late veneer, that are more likely to contribute to the OIB source, are required to have *ε*^100^Ru < 0 to lower the *ε*^100^Ru > 0 composition of the Eoarchean mantle to the present day mantle value (*ε*^100^Ru ≈ 0)^6^ and, therefore, do not have an appropriate composition. As such, the Earth’s core is at present the most viable source to explain the combined origin of positive *ε*^100^Ru and negative *μ*^182^W values observed in OIBs.

## Methods

### Samples

Samples analysed in this study comprise basalts, picrites and ultramafic cumulates associated with the Hawaii, Réunion, Galápagos and Iceland mantle plumes. Hawaiian samples with National Museum of Natural History catalogue numbers were provided by the Department of Mineral Science of the Smithsonian Institute (Data Repository). Samples from Kilauea are ultramafic cumulate samples from the 1981 drilling project on the Kilauea Iki lava lake^46^ for which W and He isotope ratios have been previously determined^5,47^. Furthermore, we determined the Ru isotopic compositions for submarine basalts and picrites from the Kama’ehuakanaloa (Loihi) volcano, for which W and He isotope data have been determined previously^1,8,22,48^. We also determined combined W and Ru isotope data for a new sample (12787) collected from the summit of the Kama’ehuakanaloa seamount. We selected samples from Kauai based on high ^3^He/^4^He ratios reported for olivine basalts from the Napali Member, formed during the shield volcanic stage of Kauaii^49^. For some historic samples from Kauaii coordinates were not available^50^. Locations of these samples were determined based on sample descriptions from the Smithsonian sample catalogue and found to be situated well within the outcrop area of the Napali Member. Tungsten isotope data were determined for all samples from Kauaii and further Ru isotope data for samples 11k and K-8. We also provide the Ru isotopic composition of a sample from Oahu (KOO-17a), for which ^3^He/^4^He values have been previously determined^51^. Extra Ru isotope data were determined for a 17.5 Ma picritic komatiite that formed as a part of the aseismic ridge of the Galápagos hotspot track accreted to the coast of Burica Peninsula in Costa Rica^52^. W isotope data for this sample have been previously determined^53^. We conducted W isotope measurements for two basalts from Fernandina (Galápagos) to augment the existing W dataset for this island. The basalts were subaerially deposited during the 1995 (Fe 15-23) and 2009 (Fe 15-12) eruptions. We determined the W and Ru isotopic composition of a basalt sample from the 1977 eruption of the Piton de la Fournaise (REU 14-31), and the W isotope composition for two samples from La Réunion, for which W isotope data have been previously reported^2^. Last, we determined the Ru isotopic composition for a set of high ^3^He/^4^He picrites from Baffin Island, representing the oldest volcanic expressions of the Iceland plume^21^.

Besides lavas from OIB settings, we analysed a variety of other mafic and ultramafic samples to validate our analytical procedure and further constrain the composition of the upper mantle. To compare measurement uncertainties and reproducibility to previous studies, we analysed the commercially available pegmatitic pyroxenite OREAS 684 (Ore Research & Exploration Pty Ltd) sourced from Merensky Reef ores from the 2.05 billion-year (Gyr) Bushveld Complex. Sample IR1513 is a carboniferous picrite collected from the Gönnern quarry in Hessia, Germany. These submarine volcanic rocks were probably deposited in a back-arc environment and are characterized by trace element compositions resembling modern E-MORB^54^. Sample EIF-1 is a composite sample of lherzolite-harzburgite mantle xenoliths from the West Eifel volcanic field. The 3.7-Gyr-old ultramafic meta-dunite sample TM220719 1A has been sampled from a dunite lens in the NW-arm of the Isua supracrustal belt and has been variably interpreted as thrust-emplaced mantle rocks^55^ or cumulates^56,57^. The sample location is equivalent to sample 194907 previously constrained for Ru isotopes in ref. ^6^.

### Sample preparation

**Extended Data Fig. 2:**
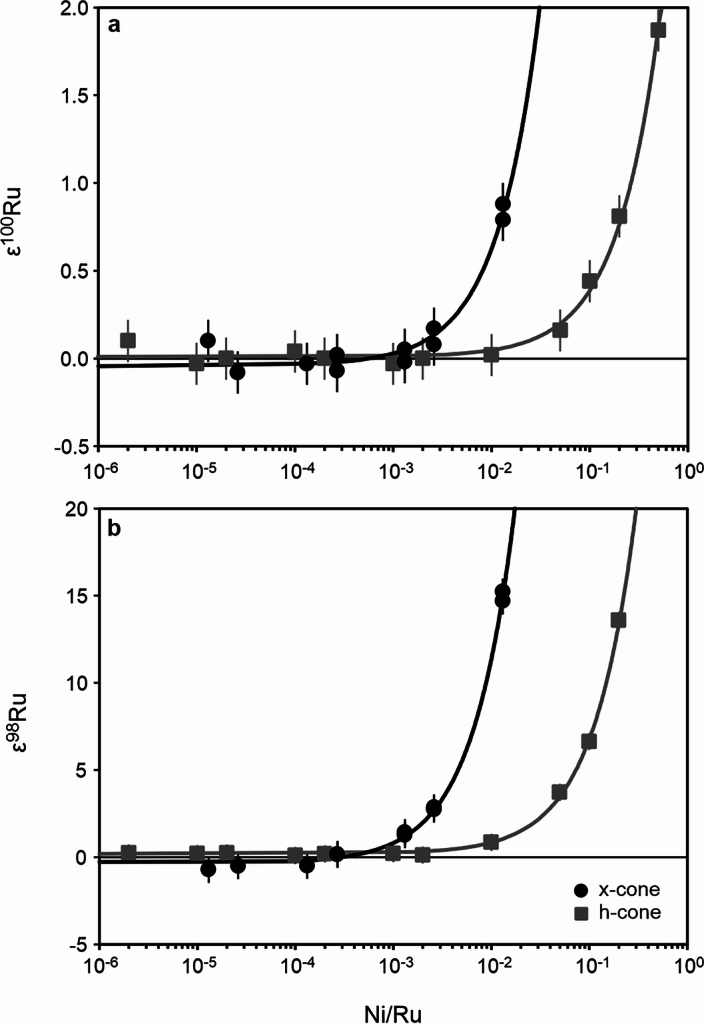
ε^100^Ru and ε^98^Ru of 40 ng/g Ru solutions doped with Nickel, illustrating the difference in NiAr formation rate for x- and h-type skimmer cones during sample measurement. Black circles are data from measurements using x-type skimmer cones. Grey squares are data from^6^ using h-skimmer cones. Uncertainty is defined by the 2 SD of repeated measurements of OREAS 684. No influence of NiAr on the Ru isotopic composition can be observed for analytes with Ni/Ru <10^−4^. Error bars represent the external reproducibility of a reference material in 2σ. Source data

**Extended Data Fig. 3:**
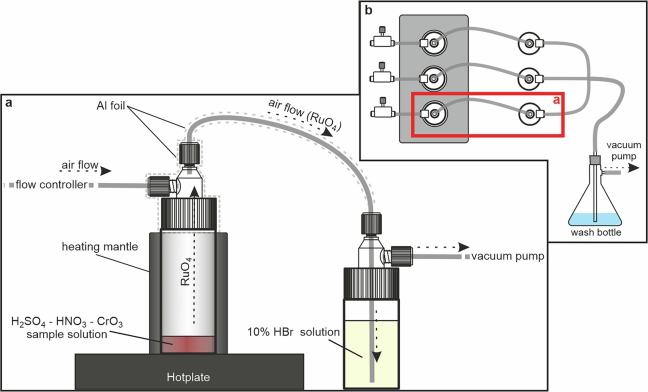
Schematic representation of the steam distillation procedure for purifying Ru. (a) Side view of a single distillation unit. The PFA tubing in the oxidation chamber was inserted no more than 3 mm into the beaker to avoid sample contamination. (b) top view on a hotplate with three distillation units. The airflow in each unit is separately controlled by a flow controller. All units are interconnected via PFA tubing and connected to the wash bottle via silicone tubing. Panel **a** was adapted from ref. ^69^ with permission from the Royal Society of Chemistry under a Creative Commons licence CC BY 3.0.

Whole rock material was processed by removing the weathering crust using a metal saw. Larger samples were then cut into smaller than 2-cm-thick slabs. All saw marks were removed by polishing with silicon-carbon sandpaper on a polishing table and subsequently washed with water. For small and brittle sample pieces the cutting process was omitted. Samples were then wrapped in several layers of plastic foil to avoid metal contamination during subsequent crushing using a hydraulic press. The samples were crushed until all pieces were smaller than 5 mm and subsequently milled to produce a fine powder (less than 125 μm) using an agate ball mill. The Baffin Island picrites were instead crushed in a tungsten-carbide jaw crusher before milling in an agate ball mill. The basic laboratory procedures for Ru isotopes are described elsewhere^6,27,58^. The overall low Ru concentration of the samples (0.25–1.7 ng g^−1^) as well as low and inconsistent Ru procedural yields reported in previous studies (that is, 20–90% ref. ^23^; less than 60–80%, ref. ^58^; 40–80%, ref. ^6^ and 30–80%, ref. ^59^) necessitated substantial improvements to the overall procedure and measurement setup published in the recent literature. Throughout the study, double-distilled acids (Savillex, DST-1000) and 18.2 MΩ cm^−1^ water (Merck Millipore) were used to process the samples. Commercially available high-purity HBr (Romil-UpA) was used during the early stages of the study. Yet, we decided to switch to HBr purified in-house (distilled five times) to improve Mo blanks (data in Extended Data Table 1 marked accordingly). All extra reagents are commercially available and a detailed list is provided in Extended Data Table 3. For each sample, 20–240 g of sample powder was preconcentrated using a nickel sulfide fire assay (NiS-fa). For the NiS-fa 20-g aliquots of sample powder were added to 130-ml porcelain crucibles with the addition of 26 g of anhydrous borax, 14 g of sodium carbonate, 1 g of nickel and 0.75 g of sulfur. The samples were thoroughly homogenized using a glass stirring rod, placed in a preheated muffle furnace and fluxed at 1,020 °C for 90 min. The samples were then removed from the furnace and quenched in room-temperature air. A NiS bead, usually weighing between 1.43 and 1.47 g was removed from the glass matrix using alumina mortar and pestle. Without crushing, each bead was dissolved in 20 ml of concentrated HCl at 130 °C for 12 h. After complete drying, this step was repeated using 25 ml of concentrated HCl and 30 ml of 6 M HCl. During the last step, 50 μl of H_2_O_2_ were added to the sample several times until no residues were visible. This usually required a total of 50–250 μl of H_2_O_2_. The samples were fully dried down followed by 10 ml of H_2_O, added in two consecutive steps. The sample is finally dissolved in 20 ml 0.2 M HCl and purified using the cation exchange procedure^6^. For this, each digestion was split and loaded on two separate glass columns (1 cm inner diameter; 30 cm length) filled with 20 ml of AG 50 W-X8 (100–200 mesh, BioRad) cation exchange resin. The platinum group elements were separated from the Ni matrix using 14 ml of 0.2 M HCl. The latter was subsequently removed in five column volumes of 6 M HCl. Split samples were recombined and evaporated at 100 °C. The samples were dissolved in 4 ml of 0.2 M HCl and passed through 2-ml cation exchange columns (BioRad Poly-Prep). This cleanup chemistry was repeated a second time to ensure the quantitative removal of Ni. This procedure is imperative because NiAr interferences later during mass spectrometric analysis will prohibit precise quantification of Ru isotope ratios during the measurement procedure for analyses with Ni/Ru greater than 1 × 10^−4^ (Extended Data Fig. 2). Finally, the Ru fraction was purified using a distillation procedure. Here, the Ru fraction was dissolved in 1 ml of concentrated H_2_SO_4_ and 1.24 ml of H_2_O and transferred into a 30-ml Savillex beaker fitted with a 33 mm impinger closure (oxidation vessel). To each oxidation vessel 1 ml of 0.457 g ml^−1^ aqueous CrO_3_ solution and 0.1 ml of concentrated HNO_3_ were added. The beaker was connected to a 20-ml Savillex beaker with impinger closure (reduction vessel) through a one-eighth of an inch diameter PFA (perfluoroalkoxy) tubing. The reduction vessel was filled with 10 ml of 10% HBr solution and connected to a chemical-resistant diaphragm vacuum pump (Rocker Chemker 411) through silicone tubing. A 2-l washing bottle filled with a weak KOH solution was interconnected to reduce the amount of acid vapour in the vacuum pump. With this setup, up to three samples could be distilled at the same time. The vacuum pump was set to a constant vacuum of 550 mmHg. A flow controller (PN 10 one-eighth of an inch diameter EM Technik) was connected upstream of the oxidation vessel to control the airflow. The controller was set so that a constant stream of air bubbled through the reduction beaker at a rate of 300–350 bubbles per min. The oxidation beaker was uniformly heated from the bottom to the base of the impinger closure in a PFA-coated aluminium block. A schematic of the distillation setup is provided in Extended Data Fig. 3. To ensure an even temperature distribution, the closure, as well as the transfer tubing, were wrapped in aluminium foil and the entire hotplate was covered with an extra layer of foil. The oxidation vessel was heated to 90 °C for 3 h. The vacuum was slowly released on completion of the distillation. The HBr solution containing the samples was evaporated for 3.5 h at 130 °C. Subsequently, 1 ml of 2 M HCl was added to each sample and evaporated at 100 °C. Finally, appropriate amounts of 0.28 M HNO_3_ were added to obtain a 40 μg g^−1^ Ru solution for analysis. We obtained a total average procedural blank of 428 ± 67 pg based on five blank samples passed as unknowns through the entire chemical purification procedure. For samples with the lowest Ru concentrations processed in our study (0.25 ng g^−1^) this equates to a blank contribution of 7.6%. Distillation yields were determined on 1‰ aliquots taken before and after distillation to be 60–100% with an average of 87% based on 109 individual distillations and are significantly higher than those reported in previous studies (above). The improvement in distillation yield can be attributed to two main factors. First, the volatile RuO_4_ rapidly decomposes under hot humid conditions to form solid, insoluble RuO_2_ (refs. ^60,61^). In this context, we found significantly improved distillation yields when oxidizing and volatile acids were added to the oxidizing vessel^62^. The addition of HNO_3_ and resulting HNO_3_ vapour leads to significantly improved stability of volatile Ru species when compared to H_2_O vapours^61^. Second, uniform heating and high extraction volumes of the vacuum pump inhibit any condensation in the oxidation vessel. Initial testing at lower extraction rates with no thermal insulation from Al foil fractions of Ru could frequently be detected in water droplets deposited in the closure of the oxidation vessel.

### Ru isotope measurement

The Ru isotopic compositions of samples and reference materials were determined on a Thermo Fisher Scientific Neptune Plus multi-collector inductively coupled plasma mass spectrometer (MC–ICP–MS) at the Department of Geochemistry and Isotope Geology of the University of Göttingen. A Teledyne Cetac Aridus III desolvating nebulizer equipped with the QuickWash3 accessory was used as the sample introduction system with a fixed capillary PFA nebulizer tip (ESI, uptake rate 68 μl min^−1^). All measurements were performed using Ni X-type skimmer cones and standard Ni sampler cones. This yielded ion beam intensities ranging from 1 × 10^−10^ to 1.35 × 10^−10^ A for a 40 ng g^−1^ Ru solution for oxide formation rates of Ce/CeO less than 5% (typically 2–3%). Use of X-type skimmer cones increased the formation rate of NiAr (Extended Data Fig. 2). Ni/Ru ratios were monitored before measurement and found to be below 10^−4^ for all samples. The Ru isotope measurements were conducted in static mode with simultaneous measurement of all stable Ru isotopes and masses 97 and 105 to monitor isobaric mass interferences of Mo and Pd. Faraday cups were connected to 10^11^ Ω feedback resistors for most masses. Amplifiers with 10^12^ Ω resistors were used for mass 98 and 10^13^ Ω resistors for masses 97 and 105. Each sample measurement was bracketed by a 40 ng g^−1^ Ru single-element reference solution (CPI International). Measurements of sample and standard solutions comprised 100 measurement cycles with 8.4 s of integration time each. For a single analysis, about 45 ng of Ru were required. Sample and standard measurements were preceded by on-peak baseline measurements of 0.28 M HNO_3_ consisting of 40 cycles with 8.4 s of integration time. Raw intensity data were corrected offline for baseline and instrument-induced mass fractionation using a Python-based script. The script first subtracts on-peak baseline measurements from measured intensities. Afterwards, mass fractionation was corrected using the exponential law relative to a constant ^99^Ru/^101^Ru value of 0.7450754 (ref. ^63^), Interference corrections for Pd and Mo were applied, followed by a 2*σ* outlier test. Isotope variations were then calculated as *ε*^i^Ru = ((^i^Ru/^101^Ru)_sample_/(^i^Ru/^101^Ru)_standard_ − 1) × 10,000 against the CPI bracketing standard. The external reproducibility (2*σ*) was determined through repeated analysis of certified reference material OREAS 684 (total of 72 single measurements, 13 individual digestions). This yielded an external reproducibility (2 s.d.) of ±0.55 for *ε*^96^Ru, ±0.76 for *ε*^98^Ru, ±0.13 for *ε*^100^Ru, *±*0.18 for *ε*^102^Ru and ±0.42 for *ε*^104^Ru (Supplementary Table 1). The external reproducibility of individual isotopes is comparable to those of previous studies^6^. Variably high *ε*^96^Ru values in standards and reference materials are due to isobaric interferences of ^96^Zr, which could not be monitored during sample measurement and therefore remain uncorrected.

### Ruthenium concentrations

In addition to Ru isotope data, we provide an estimate of the Ru concentration of the samples measured in Extended Data Table 1. Ru concentrations were determined on 1‰ sample aliquots of individual NiS digestions, taken after the first cation chemistry. Aliquots were diluted in 0.5 ml of 0.28 M HNO_3_ and measured alongside a set of four gravimetrically calibrated HSE standards on a Thermo iCAP quadrupole ICP-MS at the University of Göttingen. The Ru concentrations provided are purely informational values as the samples will have probably already lost Ru during NiS digestion and cation chemistry and only provide an estimate of the Ru concentration. The uncertainties provided reflect the standard deviation of repeated digestions of the same sample. Samples for which no error is provided have only been digested once and were found to have insufficiently low Ru concentrations for further isotope analysis.

### W isotope procedures

The W isotopic compositions of samples and reference materials were determined on a Thermo Fisher Scientific Neptune Plus MC–ICP–MS at the Department of Geochemistry and Isotope Geology of the University of Göttingen. The laboratory and measurement procedures have been described in detail elswhere^53^. The sample preparation procedures for W introduce varying effects on ^183^W (refs. ^64,65^). Although negative *μ*^183^W values measured in this study indicate a similar effect on our samples (Extended Data Table 1), we used ^186^W/^184^W ratios to correct for mass bias to avoid any influence of the ^183^W effect on *μ*^182^W values. We have previously shown that normalization to ^186^W/^183^W can still produce valid *μ*^182^W values if correction factors are applied^53,65^, indicating that no unaccounted effects influence the isotopic composition of W. W isotope ratios are reported as *μ*^182^W, which is defined as (^182^W/^184^W_sample_ / ^182^W/^184^W_NIST3163_ − 1) × 1,000,000.

For each sample, between 3 and 12 individual measurements were conducted based on the sample concentration. The external reproducibility was calculated by repeated measurements of in-house reference basalt ‘Me21’, which were measured at the beginning and the end of every measurement sequence. The external *μ*^182^W reproducibility (2 s.d.) is 4.8 ppm for basalt Me21 (*n* = 41) for triplet measurements. For sextuplet analysis, the reproducibility was improved to 3.6 ppm for basalt Me21 (*n* = 14) and to 3.3 ppm for any sample analysed nine times or more (*n* = 8). All *μ*^182^W data measured in this study are provided in Extended Data Table 1.

Furthermore, we reanalysed sample KK 25-4 from the Kama’ehauakanaloa (Loihi) submarine volcano (Hawaii)^8^ and samples REU 1001-053 and REU 1406-24.9a (La Réunion)^2^, for which data have previously been reported. For sample KK 25-4 our *μ*^182^W value of −16.3 ± 3.6 is in good agreement with the previously reported value of −13.1 ± 6.7 (ref. ^8^). Samples REU 1001-053 and REU 1406-24.9a have *μ*^182^W values of −1.5 ± 3.6 and −8.8 ± 3.6, respectively. Their isotopic compositions are significantly less anomalous than those previously reported (*μ*^182^W = −15.7 ± 3.2 and −20.2 ± 5.1, respectively^2^). Discrepancies between W isotope ratios measured by Neptune MC–ICP–MS and those measured by thermo ionization mass spectrometry have been reported in the past^8,66^. Indeed, *μ*^182^W data for La Réunion reported in further studies indicate more restricted *μ*^182^W variations between 0 and −10 (refs. ^45,67^). Our new data confirm that *μ*^182^W anomalies in basalts from La Réunion probably do not significantly exceed values of −10.

We also constrained W isotope data for two new samples from Fernandina (Galápagos) where some of the most negative *μ*^182^W values have been previously reported (*μ*^182^W = −22.7)^1^. The new samples, Fe 15-12 and Fe 15-23, have *μ*^182^W of −14.7 ± 3.6 and −17.5 ± 3.6, respectively. They confirm the anomalous W isotope composition of Fernandina basalts, however, they cannot be directly compared to those previously analysed due to unknown age relationships. For this study, we define the range of *μ*^182^W variations in the global OIB record by the most anomalous sample independently constrained in many studies. As such we assume a range of *μ*^182^W = 0 to −20 based on the sample OFU-04-14 from Samoa (*μ*^182^W = −20.2 ± 3.9 and −17.3 ± 4.5, refs. ^5,23^).

## Online content

Any methods, additional references, Nature Portfolio reporting summaries, source data, extended data, supplementary information, acknowledgements, peer review information; details of author contributions and competing interests; and statements of data and code availability are available at 10.1038/s41586-025-09003-0.

## Supplementary information

Supplementary Table 1 This file contains the Ru isotope data of reference materials measured throughout this study. This includes data for individual analysis of certified reference material OREAS 684 and in-house reference samples EIF-1, 1513 and TM220719 1A.

## Source data

Source Data Fig. 1

Source Data Fig. 2

Source Data Fig. 3

Source Data Extended Data Fig. 1

Source Data Extended Data Fig. 2

## Figures and Tables

**Extended Data Table 1:**
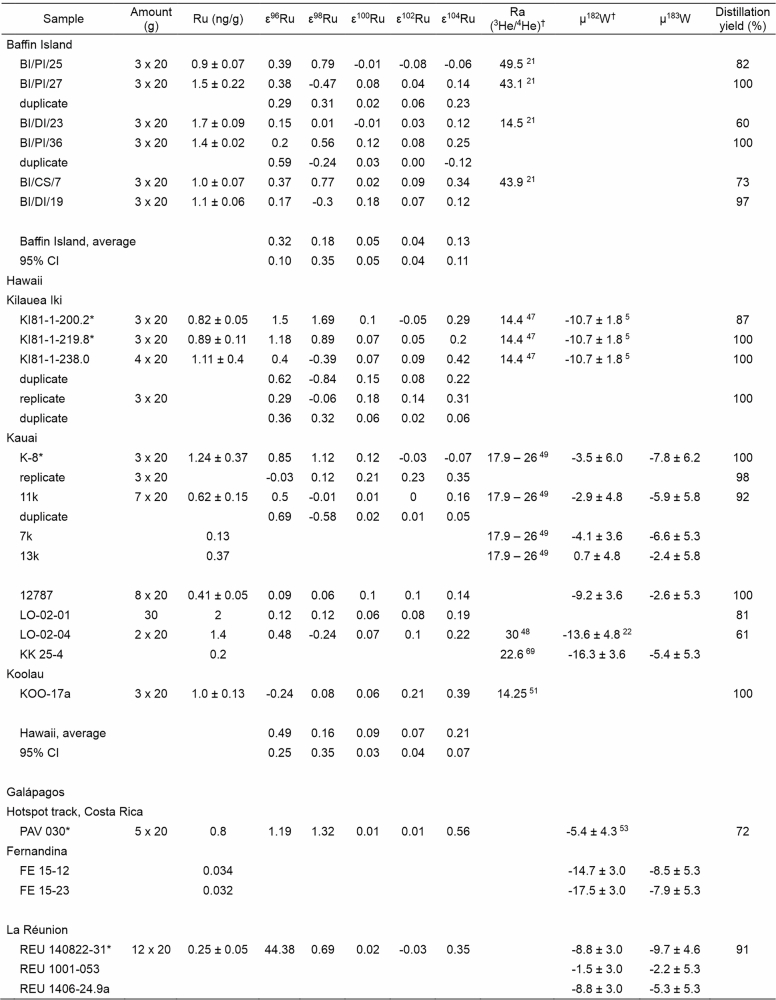
Ru and W isotope data for oceanic basalts

Uncertainties for individual measurements (2*σ*): ε^96^Ru = 0.55, ε^98^Ru = 0.76, ε^100^Ru = 0.13, ε^102^Ru = 0.18, ε^104^Ru = 0.42. ‘Replicate’ indicates that data were obtained by repeated digestion of a powder aliquot, while ‘duplicate’ is a repeated measurement of the same purified sample fraction. See Supplementary Table 1 for data of individual measurements of geological reference material samples.

* Samples distilled with commercially available HBr (ROMIL Ltd., UpA grade); all other samples distilled with HBr purified in-house (see Methods for detail).

^†^ W and He isotope data for the sample or the locality originally reported in the given reference.

**Extended Data Table 2:**
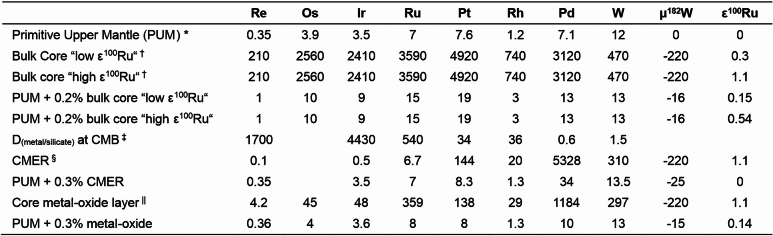
HSE concentrations (ng/g), μ^182^W and ε^100^Ru calculated for terrestrial reservoirs

* Data from^42,70,71^^†^ Calculated after^29^ using CI chondrite data from^72,73^^‡^ Calculated using equations 19 and 20 from^35^ at CMB conditions as described in^1^.

^§^ After^1^. ^||^ Based on the partitioning of metal liquid without oxygen and metal liquid with 5% O^34^.

**Extended Data Table 3:**
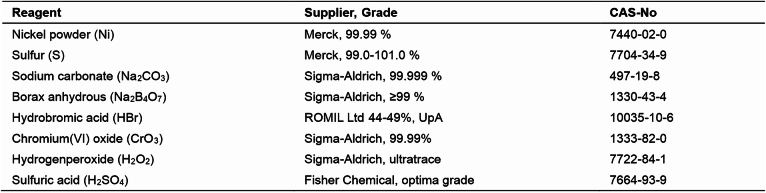
List of commercially available reagents

## Data Availability

All data produced in this study are available in Extended Data Table 1 and Supplementary Table 1 and are archived in the DIGIS geochemical data repository available at 10.5880/digis.2024.003. Source data are provided with this paper.
